# Milk and Blood Pharmacokinetics of Tylosin and Tilmicosin following Parenteral Administrations to Cows

**DOI:** 10.1155/2014/869096

**Published:** 2014-08-06

**Authors:** Tulay Avci, Muammer Elmas

**Affiliations:** ^1^Department of Toxicology, Veterinary Control Institute, 42080 Konya, Turkey; ^2^Department of Pharmacology and Toxicology, Faculty of Veterinary Medicine, University of Selcuk, 42003 Konya, Turkey

## Abstract

The aim of this study is to determine the pharmacokinetics of tylosin and tilmicosin in serum and milk in healthy Holstein breed cows (*n* = 12) and reevaluate the amount of residue in milk. Following the intramuscular administration of tylosin, the maximum concentrations (*C*_max_) in serum and milk were found to be 1.30 ± 0.24 and 4.55 ± 0.23 *µ*g/mL, the time required to reach the peak concentration (*t*_max_) was found to be 2nd and 4th h, and elimination half-lives (*t*_1/2*β*_) were found to be 20.46 ± 2.08 and 26.36 ± 5.55 h, respectively. Following the subcutaneous administration of tilmicosin, the *C*_max_ in serum and milk were found to be 0.86 ± 0.20 and 20.16 ± 1.13 *µ*g/mL, the *t*_max_ was found to be 1st and 8th h, and the *t*_1/2*β*_ were found to be 29.94 ± 6.65 and 43.02 ± 5.18 h, respectively. AUC_milk_/AUC_serum_ and *C*_max-milk_/*C*_max-serum_ rates, which are indicators for determining the rate of drugs that pass into milk, were, respectively, calculated as 5.01 ± 0.72 and 3.61 ± 0.69 for tylosin and 23.91 ± 6.38 and 20.16 ± 1.13 for tilmicosin. In conclusion, it may be stated that milk concentration of tylosin after parenteral administration is higher than expected like tilmicosin and needs more withdrawal period for milk than reported.

## 1. Introduction 

Tylosin (tylosin A) was first derived in 1960 by Mac Fuire from* Streptomyces fradiae* cultures. Tylosin exists in several forms, including the minor constituents desmycosin (tylosin B), macrocin (tylosin C), and relomycin (tylosin D), and is known for its metabolites lactenocin (tylosin L), 5-O-mycaminosyltylonolide (OMT), and demycinosyl-tylosin (DMT). Tylosin exerts bacteriostatic effect on* Mycoplasma* species and Gram-positive bacteria. It is well absorbed when administered by oral and parenteral routes and is eliminated from the body slowly. Tylosin binds to serum and milk proteins at rates of 25–47% and 15%, respectively [1, 2]. In most animal species, the half-life (*t*_1/2_) of tylosin is 3-4 h and the volume of distribution (*V*_*d*_) ranges between 1 and 7 L/kg. Tylosin passes readily into milk and its concentrations in milk may be five times higher than its plasma concentrations. Tylosin does not undergo major modification in the body and is excreted mainly in bile and milk and partly in urine [3]. The unmodified form of tylosin passes into milk and eggs. As reported by the European Medicines Evaluation Agency (EMEA), the maximum residue limit (MRL) established for tylosin in cow's milk is 50 *μ*g/kg. The withdrawal periods for meat and milk, in the event of the parenteral administration of tylosin to cattle, sheep, and goats, are 28 days and 8 milkings, respectively.

Tilmicosin is a 16-membered ring, semisynthetic macrolide antibiotic, with a chemical structure of 20-deoxo-20-(3,5-dimethylpiperidin-1-yl) desmycosin [4]. This drug is used in both cattle [5] and calves [6], for prophylactic and therapeutic purposes, against pneumonia caused by* Pasteurella multocida*,* Pasteurella haemolytica*,* Actinobacillus pleuropneumonia*, and* Mycoplasma* species [7]. Tilmicosin is preferably administered by subcutaneous (SC) route, but it may also be administered by intramuscular (IM) route. When administered subcutaneously or intramuscularly, tilcomisin reaches its peak plasma concentration (ranging between 0.8 and 1.7 *μ*g/mL when administered at normal doses) within approximately 60 min. Tilmicosin accumulates particularly in the lungs, and its pulmonary concentration may be 50 to 60 times higher than its plasma concentration. It is converted mainly into desmethyl tilmicosin in the body and is excreted mainly in the bile, either unmetabolized or in the form of its metabolites. Tilmicosin may also be eliminated in the urine [3, 8]. As reported by the EMEA, the MRL established for tylosin in cow's milk is 50 *μ*g/kg. In cattle, when administered parenterally, the withdrawal period for meat (applied prior to slaughter) is 60 days.

This study was aimed at the measurement of the levels of tylosin and tilmicosin, which are both macrolide antibiotics, in positive serum and milk samples obtained by the administration of 17.5 mg/kg bw of tylosin by IM route and 10 mg/kg bw of tilmicosin by SC route to clinically healthy Holstein cattle, following the method validation of high-pressure liquid chromatography-UV (HPLC-UV). It was also aimed at determining certain pharmacokinetic parameters and at assessing antibiotic residues in milk.

## 2. Materials and Methods

### 2.1. Experimental Animals

The study was carried out on 12 healthy dairy cattle (Holstein, 350–400 kg, 3-4 years, mean daily milk yield of 18–22 kg), the milk somatic cell counts of which were determined to be ≤500.000 by microscopy. To prevent the administration of any drugs, the animals were maintained under the same management and feeding conditions for 1 month. This study was approved by the Ethics Board of Selcuk University, Faculty of Veterinary Medicine (no. 2007/064).

### 2.2. Drug Administration and Sampling

The animals were randomly allocated to 2 groups, each composed of 6 cattle. The first group was administered with 10 mg/kg bw of tilmicosin (Micotil 300, injectable) into the dorsolateral region of the neck by SC route, while the second group was administered with 17.5 mg/kg bw of tylosin (Tylan 200, injectable) into the dorsolateral region of the neck by IM route.

The antibiotics were administered prior to the morning milking, between 06.00 and 07.30 a.m. Blood samples (10 mL) were collected from the jugular vein into sterile vacutainers (in glass centrifuge tubes) prior to antibiotic administration (0) and 10, 20, and 40 min and 1, 2, 3, 4, 6, 8, 10, 12, 24, 48, 72, and 96 h after administration. Within 1 h after being collected, the blood samples were centrifuged at 3500 rpm for 15 min. The serum samples obtained were stored at −20°C until being analysed.

At each sampling interval, prior to antibiotic administration (0) and at 0.5, 1, 2, 4, 8, 12, 24, 48, 72, 96, and 120 h after administration, the udders were milked out by hand. Milk samples (100 mL) were collected into sterile tubes, transferred to the laboratory under cold chain conditions, and stored at −20°C until being analysed.

The serum concentrations of tilmicosin and tylosin were measured using HPLC-UV and the modified methods of Moran et al. [9] and García-Mayor et al. [10].

The milk concentrations of tilmicosin and tylosin were ascertained by means of HPLC-UV and using the modified methods of Dudrikova et al. [11], Stobba-Wiley and Readnour [7], and García-Mayor et al. [10]. Specificity and selectivity, linearity and measuring range, recovery and accuracy, and sensitivity limit of detection (LOD) and limit of quantification (LOQ), and precision were used as performance criteria for method validation [3].

### 2.3. Pharmacokinetic Calculations

The serum/milk concentration-time curves of tylosin and tilmicosin were drawn for each animal with the aid of the WinNonlin [12] software. The pharmacokinetic model most appropriate for the interpretation of the parameters pertaining to each of the antibiotics was determined, based on the direct inspection of the individual serum concentration-time curves and the Akaike information criteria (AIC) [13]. In all of the animals, the pharmacokinetic parameters related to the serum concentrations of tylosin and tilmicosin were found to be most consistent with the two-compartment open model. The pharmacokinetic parameters obtained for milk were calculated using a noncompartmental model analysis.

The maximum concentrations of tylosin and tilmicosin (*C*_max⁡_) and the time periods within which the maximum concentrations were reached (*t*_max⁡_) were ascertained based on the observation of the serum/milk concentration-time curves of each animal. For each of the two antibiotics, the *C*_max⁡_ values were given in mean ± SD and the *t*_max⁡_ values were presented as means.

### 2.4. Statistical Analysis

All values were presented in mean ± SD. For the time parameters (*t*_1/2ab_, *t*_1/2*α*_, and *t*_1/2*β*_), the harmonic mean ± SD was calculated. For both antibiotics, the statistical differences between the serum and milk concentrations, *C*_max⁡_, and AUC parameters were evaluated using the SPSS 15.0 statistical package programme and* paired-samples t-test*. The statistical differences for *t*_1/2*β*_ were assessed with the nonparametric* Wilcoxon signed-rank test*. The within-day and between-day differences of the method for tylosin and tilmicosin were reflected in standard deviations and percentage variation coefficients. A descriptive statistical assessment was made, based on the concentrations determined in repeated analyses, and mean ± SD and percentage (%) variation coefficients were calculated. The confidence interval was 95% in all analyses. In the statistical analyses, the level of significance was accepted as *P* < 0.05.

## 3. Results

**Figure 1:**
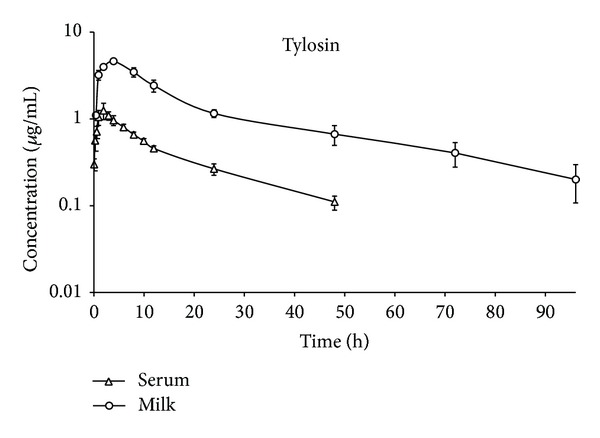
Semilogarithmic milk and serum concentration-time profiles of tylosin after the intramuscular administration of a single dose of 17.5 mg/kg (*n* = 6).

Milk/serum concentration-time curves were drawn following the intramuscular administration of a single dose (17.5 mg/kg bw) of tylosin (Figure 1). Statistical differences were determined to exist between the milk and serum concentrations of tylosin for the same sampling intervals (1, 2, 4, 8, 12, 24, and 48 h after administration) (*P* < 0.05).

**Figure 2:**
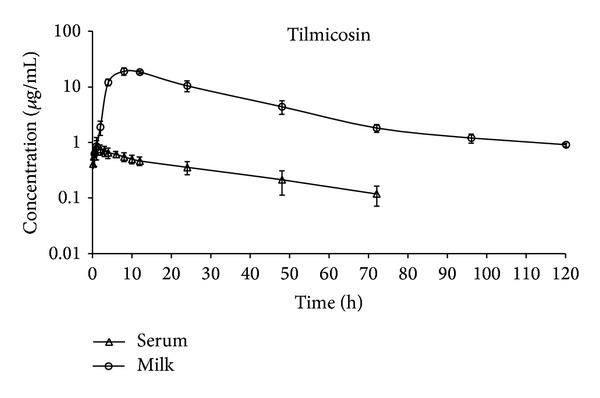
Semilogarithmic milk and serum concentration-time profiles of tilmicosin after the subcutaneous administration of a single dose of 10 mg/kg (*n* = 6).

Milk/serum concentration-time curves were drawn following the subcutaneous administration of a single dose (10 mg/kg bw) of tilmicosin (Figure 2).

It was determined that statistically significant differences existed between the milk and serum concentrations of tilmicosin measured at 2, 4, 8, 12, 24, 48, 72, and 96 h after administration (*P* < 0.05).

Pharmacokinetic parameters for tylosin and tilmicosin in serum and milk were calculated in Holstein cattle using a two-compartment open model and a noncompartmental analysis model (Table 1).

## 4. Discussion

Following IM administration, tylosin was detected from the 10th min up to 48 h after administration in the serum and from the 30th min up to 96 h after administration in the milk. These results were found to be in parallel with those reported in previous research carried out in cattle [2, 14, 15]. Following SC administration, tilmicosin was detected from the 10th min up to 72 h after administration in the serum and from the 30th min up to 120 h after administration in the milk. These results were similar to those previously reported in cattle [16] and goats [17]. It was ascertained that, following the IM administration of tylosin and the SC administration of tilmicosin, the serum concentration-time curves of both antibiotics were consistent with the two-compartment open model (Figures 1 and 2). Similarly, while the two-compartment open model has been used for calculations in previous research [16–21], it has been observed that, in studies in which a consistency analysis (based on direct observation and AIC) was not performed [8, 22–24], the noncompartmental model was used.

In the present study, following IM administration, tylosin was determined to have reached its serum maximum concentration (1.30 ± 0.24 *μ*g/mL) by the 2nd h following administration (Table 1). Similar results have been reported in a previous study in cattle [2].

The elimination half-life (*t*_1/2*β*_) of tylosin in serum was determined to be 20.46 ± 2.08 h (Table 1). This is longer than the values previously reported for other animal species (calf 2.24 h, buffalo 2.40 h, camel 2.73–3.71 h, pig 3.01–3.88 h, sheep 2.3–6 h, and goat 5 h) [15, 21, 24, 25]. The variability of the elimination half-lives of this antibiotic in different animal species is attributed to the anatomical and physiological differences between these species as well as to the differences in the formulation of the drug [25]. The long elimination half-life of tylosin in cattle demonstrates that its penetration level is low and its retention period in the tissues is long. These results are in compliance with those reported in previous research in cattle [2, 15], goats [20, 21], and sheep [25].

In the present study, following SC administration, tilmicosin was determined to have reached its serum peak concentration (0.86 ± 0.20 *μ*g/mL) by the 1st h following administration (Table 1). Similar to the results obtained in the present study, the serum peak concentrations of tilmicosin in sheep, cattle, calves, goats, and chickens were reported as 0.822, 0.873, 1.10, 1.56, and 1.28–2.12 *μ*g/mL, respectively, whilst the time period required for reaching the serum peak concentration was reported as 3.9, 0.5, 1, 6.39, and 4.66–5.82 h, respectively, in the same animal species [6, 16, 17, 23].

In the present study, the serum elimination half-life (*t*_1/2*β*_) of tilmicosin was ascertained to be 29.94 ± 6.65 h (Table 1) and this result was observed to comply with previous literature reports (cattle 29 h, goats 29.3 h, sheep 33 h, chickens 30.18–45.0 h, and foals 18.4 ± 10.7 h) [16, 17, 22, 23, 26]. Despite the physiological and morphological differences of chickens from other animal species, the pharmacokinetic parameters determined for tilmicosin in chickens display similarity to those determined in ruminants. This could be attributed to the fact that a larger part of this drug is not metabolized [16].

After administrations, tylosin and tilomicosin were first detected in milk at the first sampling interval. At all sampling intervals, the milk concentrations of the antibiotics were significantly higher than their serum concentrations (*P* < 0.05, Figures 1 and 2). These results complied with those previously reported for tylosin [1, 2, 20, 25] and tilmicosin [17]. The high concentrations of both antibiotics reached in the milk following extravenous administration could be explained by nonionic passive diffusion [2].

The AUC_milk_/AUC_serum_ and *C*_max-milk_/*C*_max-serum_ ratios of both of the antibiotics are indicators of the passage of them into the mammary glands following systemic administration in lactating dairy cattle [25].

Following IM administration, the AUC_milk_/AUC_serum_ and *C*_max-milk_/*C*_max-serum_ ratios of tylosin were calculated as 5.01 ± 0.72 and 3.61 ± 0.69, respectively. These results were found to comply with those reported by Ziv and Sulman [1] in cattle and sheep (the AUC_milk_/AUC_serum_ ratio was reported as 3.5 and the *C*_max-milk_/*C*_max-serum_ ratio was reported as 2.5), but they were observed to be lower than those reported by Al-Wabel [25] (the AUC_milk_/AUC_serum_ ratio was reported as 29.5 and the *C*_max-milk_/*C*_max-serum_ ratio was reported as 11.8).

Following SC administration, the AUC_milk_/AUC_serum_ and *C*_max-milk_/*C*_max-serum_ ratios of tilmicosin were calculated as 23.91 ± 6.38 and 20.16 ± 1.13, respectively. In a previous study carried out in goats [17], the AUC_milk_/AUC_serum_ ratio was reported as 12.0 ± 0.17 and the *C*_max-milk_/*C*_max-serum_ ratio was reported as 7.33 ± 0.13.

In the present study, following IM administration, the *C*_max⁡_, *t*_max⁡_, and *t*_1/2*β*_ values of tylosin in milk were determined as 4.55 ± 0.23 *μ*g/mL, 4 h, and 26.36 ± 5.55 h, respectively (Table 1). In parallel with the maximum milk concentrations determined for tylosin in the present study, previous research demonstrated the maximum milk concentrations of tylosin as 6.22 *μ*g/mL in cattle, 6.68–7.41 *μ*g/mL in sheep, and 6.9 *μ*g/mL in goats; and the time required to reach these concentrations as 6 hrs, 7–4.5 h, and 6 h in the same animal species, respectively [1, 20, 25].

In the present study, following SC administration, the *C*_max⁡_, *t*_max⁡_, and *t*_1/2*β*_ values of tilmicosin in milk were ascertained as 20.16 ± 1.13 *μ*g/mL, 8 h, and 43.02 ± 5.18 h, respectively (Table 1). These findings were observed to display similarity to those reported in a previous study [17]. The findings obtained in the present study demonstrated that, shortly after being administered, tylosin and tilmicosin reached high concentrations in the milk and were eliminated from the body slowly.

The pharmacokinetic parameters determined for tylosin and tilmicosin in the present study showed that these drugs remain in milk and the body tissues for a long time period. When administered at the treatment dose (10 mg/kg bw) by IM route, the withdrawal period of tylosin in milk is 8 milkings and the MRL established for tylosin in milk is 50 *μ*g/kg [27, 28]. In the present study, tylosin was administered to cattle by IM route and at a dose of 17.5 mg/kg bw. The mean tylosin concentration determined in the samples taken at 96 h after administration (0.20 ± 0.09 *μ*g/mL) was found to be above the MRL. In view of the half-life of tylosin in milk (26.36 ± 5.55 h), the withdrawal period established for milk was considered to be inadequate in ensuring the elimination of the drug.

The use of tilmicosin in lactating dairy cattle is prohibited. However, owing to a long retention period in the mammary glands and a long elimination period from the body having been determined for the SC administration of the drug, this antibiotic is known to be used illegally for the treatment of mastitis [17]. The EMEA has reported the encounter of such illegal use of tilmicosin in Europe and has also drawn attention to the lack of control of these cases [29]. The MRL in milk established for the administration of the treatment dose of tilmicosin (10 mg/kg bw) by SC route is 50 *μ*g/kg [27, 30]. The mean tilmicosin concentration determined in the milk samples taken at 120 h after administration (0.91 ± 0.07 *μ*g/mL) was found to be significantly above the MRL.

In conclusion, as it was determined that the administration of tylosin and tilmicosin (illegal use of the latter) to lactating cows requires a long withdrawal period to be applied for milk, the use of these antibiotics in dairy cattle should be carefully regulated in view of the risk of residues associated with their therapeutic administration.

## Figures and Tables

**Table 1:** Pharmacokinetic parameters of tylosin (17.5 mg/kg, IM) and tilmicosin (10 mg/kg, SC) in Holstein cows (*n* = 6) after a single parenteral administration.

Parameters	Tylosin (mean ± SD)		Tilmicosin (mean ± SD)	
	Serum	Milk	Serum	Milk
*C*_max⁡_ (*µ*g/mL)	1.30 ± 0.24	4.55 ± 0.23∗	0.86 ± 0.20	20.16 ± 1.13∗
*t*_max⁡_ (h)	2	4	1	8
AUC (*µ*g*·*h/mL)	20.95 ± 1.73	104.29 ± 12.63∗	28.42 ± 8.68	639.09 ± 65.33∗
*t*_1/2ab_ (HO) (h)	0.54 ± 0.31	—	0.21 ± 0.04	—
*α* (h^−1^)	0.26 ± 0.19	—	0.26 ± 0.09	—
*β* (h^−1^)	0.03 ± 0.003	—	0.02 ± 0.005	—
*t*_1/2*α*_ (HO) (h)	2.63 ± 2.50	—	2.68 ± 1.10	—
*t*_1/2*β*_ (HO) (h)	20.46 ± 2.08	26.36 ± 5.55∗	29.94 ± 6.65	43.02 ± 5.18∗
*V*_*d*_ (L/kg)	20 ± 0.9	—	15.56 ± 2.95	—
*K*_01_ (h^−1^)	1.28 ± 0.57	—	3.14 ± 0.54	—
*K*_12_ (h^−1^)	0.08 ± 0.07	—	0.07 ± 0.04	—
*K*_21_ (h^−1^)	0.10 ± 0.05	—	0.18 ± 0.04	—
*C*_max-milk_/*C*_max-serum_	3.61 ± 0.69		20.16 ± 1.13	
AUC_milk_/AUC_serum_	5.01 ± 0.72		23.91 ± 6.38	

*Values shown in the same row are statistically significant (*P* < 0.05).

*C*_max⁡_: maximum concentration; *t*_max⁡_: time to peak concentration; AUC: area under the curve from zero to infinity by the trapezoidal integral; *t*_1/2ab_: absorption half-life; *α*; distribution rate constant; *β*: elimination rate constant; *t*_1/2*α*_: distribution half-life; *t*_1/2*β*_: elimination half-life; *V*_*d*_; volume of distribution; *K*_01_: first-order elimination rate constant; *K*_12_ and *K*_21_: first-order rate constants for drug distribution between the central and peripheral compartments; HO: harmonic mean, SD: standard deviation.
